# How to address the barriers to meaningful adolescent involvement in health research: A qualitative study

**DOI:** 10.1111/jora.13031

**Published:** 2024-10-20

**Authors:** Azza Warraitch, Ciara Wacker, Emer Buckley, Ashling Bourke, Kristin Hadfield

**Affiliations:** ^1^ Trinity Centre for Global Health Trinity College Dublin Dublin Ireland; ^2^ School of Psychology Trinity College Dublin Dublin Ireland; ^3^ Institute of Education Dublin City University Dublin Ireland

**Keywords:** adolescent involvement, barriers, mitigation strategies, participatory research

## Abstract

The under‐involvement of adolescents in health research has been attributed to multiple barriers faced by both researchers and adolescents. Despite identifying these barriers, the literature offers few solutions, mostly from the perspective of researchers. To address this, we conducted a qualitative study to explore effective strategies to address these barriers from the perspective of both researchers and adolescents. We conducted semi‐structured interviews with adolescents (*n* = 25) and researchers (*n* = 25) from 14 countries. We included adolescents aged 10–24 years with experience of contributing to health research studies and health researchers with experience of engaging adolescents in health research. The interviews explored the mitigation strategies to commonly reported barriers to meaningful adolescent involvement for researchers and adolescents. Data were analyzed using reflexive thematic analysis. We identified three overarching strategies to address the commonly experienced barriers to adolescent involvement. First, participants suggested the need to plan for adequate resources, organizational support, capacity building, accessibility, compensation, and adolescents' safety. Second, they recommended building relationships by engaging the community, fostering trust and respect with adolescents, promoting teamwork, and maintaining transparent communication. Third, they proposed making involvement engaging for adolescents by creating a conducive environment, increasing their representation, using interesting methods, and addressing power dynamics. These findings build on the current best practices for adolescent involvement in health research by highlighting which strategies should be incorporated early on to plan for and prevent potential challenges to adolescent involvement.

## INTRODUCTION

Over the past two decades, there has been an increased demand for adolescent involvement in health research (Clarke, 2015; Patton et al., 2016). Adolescent health and well‐being are defined as “adolescents having the support, confidence, and resources to thrive in contexts of secure and healthy relationships, realising their full potential and rights” (Ross et al., 2020). Based on Ross et al. (2020) multidomain framework of adolescent health and well‐being, adolescent health research can be defined as research that aims to improve the health, nutrition, connectedness, safety and support, learning, and agency of adolescents. Some examples of this include research on adolescents' physical and mental well‐being and ill‐health, sexual and reproductive health, bullying and violence prevention, improving school belonging, or educational outcomes.

Research involving adolescents is defined as “research that is done ‘with’ or ‘by’ adolescents, not ‘to’, ‘about’ or ‘for’ them” (National Health Research Authority, 2015; Wilson et al., 2020). There are various approaches to adolescent involvement in research, including youth‐led research (such as Youth Participatory Action Research [YPAR]); engaging youth as coresearchers; and involving them as collaborators or consultants (Rouncefield‐Swales et al., 2021). Adolescents have been engaged using these approaches across disciplines such as health, community psychology, social sciences, social work, and education (Gomez & Ryan, 2016; Ozer et al., 2013 ). For example, a review of 20 years of YPAR in the United States found its application in education, social inequalities, health, violence, and safety (Anyon et al., 2018). YPAR involves young people in identifying, researching, and addressing important issues, fostering collaborative knowledge generation and actionable change (Anyon et al., 2018; Checkoway & Richards‐Schuster, 2003; Jacquez et al., 2013; Shamrova & Cummings, 2017). Similarly, adolescents have also acted as collaborators and consultants (Kirby, 2004; Shaw et al., 2011) in research across education (Domingo‐Coscollola et al., 2014; Smit, 2013), social work (Cudjoe et al., 2020), and public health (Sprague Martinez et al., 2020). Collaboration refers to an ongoing partnership with adolescents and sharing decision‐making power with them. As consultants, adolescents provide input on different aspects of the research process, which is then taken into account by researchers (Kirby, 2004). Previous research on these approaches has identified some best practices for engaging young people in research (Larkins et al., 2021; Wilson et al., 2020).

Young people should be involved in research using these different approaches for several reasons. *First*, as per Article 12 of the United Nations Convention on the Rights of the Child, children and adolescents have a right to participate in all decisions that affect them (UN, 1989). This convention has been ratified by 196 countries. The United States of America has signed the Convention but has not ratified it. *Second*, there is growing evidence that adolescent involvement in research has many benefits for the development of adolescents (Warraitch, Wacker, Bruce, et al., 2024a; Wilson et al., 2020). When adolescents are involved in research, they gain knowledge and skills (Larkins et al., 2021; Valdez et al., 2020), grow personally (Fountain et al., 2021), benefit financially (Gavine et al., 2017), advance in their careers and education (Hackett, 2019), and build relationships (Riecken et al., 2005). *Third*, adolescent involvement also positively impacts the researchers and the research process. For the research, involving adolescents makes the study more relevant to them (Wilson et al., 2020), improves recruitment (Martins, 2021), and enhances data collection (Noone et al., 2016) and analysis (van Schelven et al., 2020). Involving adolescents and young people in youth‐led projects makes it more likely for the study findings to result in social action and system‐level change (Anyon et al., 2018). Researchers also benefit from adolescent involvement by gaining new knowledge (Fountain et al., 2021) and skills (Larkins et al., 2021).

Despite the increased recognition of the importance of adolescent involvement in research, adolescents are under‐involved in health research as compared to adults (Wadman et al., 2019). For example, a recent review found that less than 1% of studies on child and adolescent health reported the involvement of adolescents as advisors (Sellars et al., 2021). This lack of meaningful adolescent engagement is striking in light of the growing demand for adolescent involvement by funders and health organizations (PMNCH, 2020).

The under‐involvement of adolescents in health research can be attributed to the barriers experienced by adolescents and researchers in the adolescent involvement process (Sellars et al., 2021; Warraitch, Lee, Bruce, et al., 2024). Researchers have reported multiple barriers to involving adolescents in research. These include organizational challenges (such as gatekeeping [Jørgensen, 2019] and limited resources [Wilson et al., 2020]), issues stemming from a lack of researchers' preparedness (Wilson et al., 2020), challenges related to the methodology of adolescent involvement (such as issues with recruiting and retaining adolescents) (Fountain et al., 2021; Preston et al., 2019), adolescent‐specific issues and challenges (such as adolescents' skills and expertise [Bradbury‐Jones et al., 2018], training [Jørgensen, 2019], and different motivation and goals [Bagnoli & Clark, 2010]), and difficulties in ensuring ethical involvement of adolescents (Cullen & Walsh, 2020). Adolescents have also reported challenges to their involvement, including lack of meaningful involvement (Bradbury‐Jones et al., 2018), reluctance to contribute to research (Sullivan et al., 2021), inaccessibility of the research process (Jørgensen, 2019), a lack of confidence in their skills (Doyle et al., 2022), managing their workload and competing demands (Griebler et al., 2017), and not being acknowledged by researchers for their work (Cullen & Walsh, 2020). Although these barriers have been identified from the perspectives of both researchers and adolescents, a recent umbrella review of reviews on adolescent involvement in health research found that, in comparison to the plethora of barriers highlighted, few mitigation strategies have been reported to address these barriers (Warraitch, Lee, Bruce, et al., 2024b). However, this may not apply to other disciplines such as social policy, which have made some progress in providing strategies for barriers to youth involvement in research (Morciano et al., 2014). In health research, previous studies on adolescent involvement, using different approaches highlighted above, have identified some best practices. However, there is a need to explore which practices can address the most frequently experienced barriers to adolescent involvement in health research (Warraitch, Lee, Bruce, et al., 2024) and to generate more evidence to understand the mechanisms through which these barriers can be addressed for both researchers and adolescents in different contexts.

Several researchers have conducted consultations and interviews with different stakeholders to explore the barriers to adolescent involvement in health research. However, these studies either focused on exploring the barriers to adolescent participation in specific areas of health such as mental health research (Faithfull et al., 2019; Wadman et al., 2019), or mental health services (Oruche et al., 2014; Radez et al., 2021; Spielvogle, 2011), or did not explore the mitigation strategies to the barriers experienced during the adolescent involvement process (Das et al., 2020; Faithfull et al., 2019; Hawke et al., 2020; Wadman et al., 2019)

Furthermore, adolescents' perspectives were rarely represented, as most studies looked at the barriers experienced by researchers in involving adolescents. This overemphasis on exploring adolescent involvement primarily through adult perspectives reinforces adultism, implying that researchers' views and experiences are more valuable than those of adolescents. To address this gap, we conducted a qualitative study with adolescents and researchers from various countries to identify strategies for mitigating common barriers faced by both groups during the process of involving adolescents in health research. Our research questions were as follows: (i) What strategies do researchers employ to mitigate the challenges faced in involving adolescents aged 10 to 24 years meaningfully in health research? (ii) What strategies do adolescents perceive to be useful to mitigate the challenges faced in their meaningful involvement in health research?

## METHODS

The research questions were addressed using a qualitative study design. Ethics approval for this study was obtained from the Health Policy and Management and Centre for Global Health Research Ethics Committee (HPM/CGH REC) at Trinity College Dublin (Application number: 04E/2022/04). This study was preregistered at Open Science Foundation (OSF) (https://doi.org/10.17605/OSF.IO/YPN4H). All study materials are available on the study OSF page (https://osf.io/ypn4h?mode=&revisionId=&view_only=).

### Positionality statement

We are a diverse team, each member bringing unique perspectives that shape our approach to studying adolescent involvement in health research. The team includes a PhD student (AW) whose work focuses on developing guidelines for adolescent involvement in health research. AW also has an understanding of the challenges faced by adolescents in resource‐limited settings, informed by her experience of working to promote mental health among school‐going adolescents in rural Pakistan. CW and EB are undergraduate psychology students, acting as adolescent coresearchers, who contributed their unique insights and lived experiences to this study. Their active involvement in various student organizations and lived experience of adolescence provide valuable perspectives on the barriers of adolescent involvement in research. AB is an Associate Professor in Psychology and Human Development, whose research focuses on child and adolescent well‐being, education, and children's rights. As a researcher and educator, AB is committed to advocating for the rights and well‐being of children and adolescents in research and practice, particularly supporting participative rights alongside protection and provision rights. KH is an Assistant Professor focusing on resilience, well‐being, and mental health of young people. Her research includes developing and evaluating interventions to promote resilience and mental health among adolescents. This background provides valuable expertise in designing strategies that effectively engage adolescents in research and address their unique needs and perspectives.

To address the influence of our positionalities, we used several reflexive practices throughout the research project. Detailed notetaking allowed us to record our thoughts and feelings, ensuring a transparent record of our reflections. Meetings among team members provided opportunities to discuss and reflect on our findings and interpretations, enhancing the credibility of our analysis. Member checking involved seeking feedback from participants to validate the accuracy and authenticity of our data and interpretations. Triangulation of viewpoints (researchers and adolescents) further strengthened the rigor of our study.

### Participants

Semi‐structured interviews were conducted with 25 adolescents and 25 researchers. Initially, we planned for a starting sample size of 25 participants, with 13 adolescents and 12 researchers. However, given that saturation was not achieved at that point, we continued interviewing participants, ultimately concluding after 50 interviews. A combination of purposive and snowball sampling techniques was used to recruit participants.

To be eligible for participation in this study, adolescents had to be aged 10–24 years inclusive, able to communicate in English, and possess experience contributing to a health research study as an advisor, consultant, collaborator, or coresearcher. A total of 25 adolescents (12 male, 13 female; age range 15–24 years, *M*_age_ = 21.1 years, SD = 2.6) were recruited from 14 countries (Cameroon, Canada, Ghana, India, Ireland, Kenya, Liberia, Netherlands, Pakistan, Rwanda, Somaliland, South Africa, United Kingdom, and United States of America). Of these, 6 adolescents were from high‐income countries, and 19 were from low‐ and middle‐income countries. We aimed to include more younger adolescents in our sample but ended up involving older adolescents (aged 15 and above) due to challenges in the recruitment process. However, many of these participants had been younger when initially being involved in different research projects. Recruitment was conducted through various channels: first, by posting flyers on social media platforms with a call for recruitment; second, by reaching out to over 200 youth health organizations in different countries and requesting them to share the recruitment call with their youth advisory groups; and third, by contacting youth health researchers in different countries and asking them to share the flyers with members of the youth advisory groups they collaborate with. Adolescent participants were compensated for their time with gift vouchers equivalent to €10. Although some of the interviewed adolescents were researchers themselves, we grouped their findings under the overall adolescents' category during the write‐up process.

To be eligible for participation in this study, researchers had to be able to communicate in English and possess experience engaging adolescents in one or more research studies as advisors, consultants, collaborators, or coresearchers. Researchers were recruited by emailing individuals known to the lead author due to their recognized experience in adolescent involvement in health research. Additionally, corresponding authors of papers included in a recently conducted umbrella review of barriers to adolescent involvement (Warraitch, Lee, Bruce, et al., 2024a) were also emailed and invited to participate in this study. A total of 25 researchers (6 male, 19 female) were recruited from 14 countries (Ethiopia, Ghana, India, Ireland, Kazakhstan, Kenya, Lebanon, Moldova, New Zealand, Nigeria, Pakistan, Tanzania, United Kingdom, and United States of America). Of these, 13 researchers were from high‐income countries, and 12 were from low‐ and middle‐income countries.

### Procedure

We used separate interview topic guides for adolescents and researchers. All interviews were conducted in English. The interview topic guides were developed by AW and KH, drawing on the findings of a recently conducted umbrella review exploring the barriers to adolescent involvement in health research (Warraitch, Lee, Bruce, et al., 2024) and a rapid review to identify currently available guidelines on adolescent involvement in health research (Warraitch, Wacker, Bruce, et al., 2024b). The interview topic guides for researchers and adolescents included questions to explore the mitigation strategies to different barriers to adolescent involvement. The interview topic guides are available on our study OSF page: (https://osf.io/ypn4h?mode=&revisionId=&view_only=). Considering the extensive list of barriers in the interview guides, our objective was to explore the mitigation strategies for as many of these barriers as feasible during each hour‐long interview session with participants. Consequently, this meant that different participants were asked about different subsets of barriers. For example, if participant A's interview addressed the first 20 barriers, participant B's interview explored the next 20 barriers on the list. This approach was adopted to ensure that we could explore mitigation strategies for all the barriers documented in the literature to some extent.

Written informed consent was obtained from all researchers and adolescents aged ≥18 years. Written informed consent was obtained from the parents of adolescents <18 years, and written assent was sought from these adolescents. Participants were interviewed by a female PhD student in Psychology. All interviews were conducted online via Zoom and were audio recorded with the written permission of participants. Adolescent interviews ranged from 35.6 min to 70.3 min (*M* = 54.0, SD = 9.6), while researcher interviews ranged from 35.52 to 80.18 minutes (*M* = 56.5, SD = 8.6). They were transcribed orthographically using the automatic caption generation function in Zoom and were proofread for accuracy and corrected by AW. Demographic characteristics are presented in Table 1.

**TABLE 1:** Sociodemographic characteristics of adolescents and researchers.

Adolescents (*n* = 25)						Researchers (*n* = 25)				
Pseudonyms	Age	Gender	Countries	Health research area	Roles	Pseudonyms	Gender	Countries	Health research area	Experience (years)
Aarti	23	Female	India	Cancer clinical trials, education access	Consultant and coresearcher	Abiy	Male	Ethiopia	Adolescent and youth sexual and reproductive health	20
Abena	24	Female	Ghana	Youth mental health and sports, dating violence	Consultant and collaborator	Aditi	Female	India	Adolescent sexual and reproductive health	4
Aisha	23	Female	Kenya	Substance use	Advisor	Aisha	Female	Kazakhstan	Public health	18
Akua	24	Female	Ghana	Climate change and health, covid vaccinations	Consultant and coresearcher	Amelia	Female	United Kingdom	Violence against women and girls	3
Ali	18	Male	Pakistan	Gender equality, schooling for autistic youth, university life and health	Consultant and coresearcher	Andrei	Male	Moldova	Adolescent health	11
Ayan	19	Female	Somaliland	Sexual and reproductive health, gender equality and education	Peer educator and coresearcher	Anjali	Female	India	Adolescent sexual and reproductive health	5
Charlotte	18	Female	United Kingdom	Mental health	Consultant and collaborator	Charlotte	Female	United Kingdom	Health and social issues	15
Ciara	16	Female	Ireland	Eating disorders, social media use	Consultant, advisor, and coresearcher	Chukwu	Male	Nigeria	Mental health	8
Daan	22	Male	Netherlands	Youth participation in health research, Mental health	Advisor	Emily	Female	United Kingdom	Youth health and social care research	20
Ekwoge	22	Male	Cameroon	Mental health	Advisor	Emma	Female	United States of America	Diabetes	15
Emily	20	Female	Canada	Bereavement, early marriage and violence prevention	Consultant and advisor	Grace	Female	United Kingdom	Substance use and social issues affecting youth	>25
Farhan	15	Male	Somaliland	Infection control	Consultant	Isabella	Female	United Kingdom	Youth disability and development	26
Fatima	20	Female	Kenya	Young caregivers of dementia patients	Collaborator	Juma	Male	Tanzania	Health and social care research	10
Jamal	24	Male	Kenya	Polio vaccinations, road safety	Consultant and coresearcher	Kwesi	Male	Ghana	Rights and well‐being of youth	1
Jean	24	Male	Rwanda	HIV research	Coresearcher	Layla	Female	Lebanon	Adolescent mental health and well‐being	10
Kadiatu	24	Female	Liberia	Mental health	Advisor	Lily	Female	United Kingdom	Adolescent mental health	6
Kofi	22	Male	Ghana	Conflict resolution	Coresearcher	Ochieng	Male	Kenya	Youth mental heath	6
Kwame	24	Male	Ghana	Mental health, biodiversity research	Consultant and advisor	Olivia	Female	United Kingdom	Health and social care research	5
Mohamed	17	Male	Somaliland	Communicable diseases	Advisor	Reha	Female	India	Well‐being of youth with disabilities	2
Nomvula	23	Female	South Africa	Climate change and health, mental health	Consultant and advisor	Sara	Female	Ireland	Youth well‐being	5
Olivier	23	Male	Rwanda	Sexual and reproductive health, mental health	Advisor and coresearcher	Selam	Female	Ethiopia	Adolescent and youth sexual and reproductive health	2
Priya	20	Female	India	Mental health	Consultant	Siobhan	Female	Ireland	Health and social care research	18
Saoirse	17	Female	Ireland	Sexual and reproductive health	Coresearcher	Sophie	Female	United Kingdom	Health and social care research	20
Thabo	23	Male	South Africa	Sexual and reproductive health	Peer educator	Sunaina	Female	Pakistan	Gender, sexual and reproductive health	8
Tyler	22	Male	United States of America	Sexual and reproductive health, universal health coverage	Collaborator and coresearcher	Zoe	Female	New Zealand	Youth mental health	20

### Analysis

We started data analysis concurrently with the data collection process. Adolescent and researcher datasets were treated as separate datasets for the purpose of the analysis since interviews with both focused on different topics. By conducting separate analyses, we ensured that the unique viewpoints and insights of both adolescents and researchers were fully captured. This approach allowed us to thoroughly examine the perspectives and experiences of each group independently before seeking convergence in the identified themes. After developing the themes for each group separately, we compared them to identify similarities and differences, ultimately concluding that the same themes emerged from the analysis of data from both groups.

We conducted a thematic analysis following the six phases of the reflexive thematic analysis as per Braun and Clarke (2006, 2019). First, AW familiarized herself with the interview transcripts by listening to the audio recordings of the interviews. Transcripts were then imported into NVivo, Version 12 (Bazeley & Jackson, 2019). AW coded all interviews using inductive coding, where codes were driven by the data. Both latent and semantic codes were used to summarize and label sentences. Data saturation was not achieved, as all interviews generated some unique codes. However, very few new codes were generated after 22–23 interviews, therefore we decided to conclude the interviews at 50 to avoid redundancy in data collection. The coding process was iterative and systematic, with AW initially coding all interviews and subsequently refining and merging similar codes. AW then categorized the final codes into preliminary subthemes and themes to summarize the key strategies for addressing the barriers to adolescent involvement in health research and then discussed these emerging interpretations with KH. Following this, the themes and subthemes were reviewed by the team and were finalized after incorporating their input. Themes for researchers and adolescents were merged during the write‐up process. This approach simplified the presentation of findings, considering the diverse roles adolescents played in research projects, ranging from consultants to coresearchers. Pseudonyms, derived from commonly used names based on the gender and country of participants, were randomly assigned to participants. The results were shared with the participants for member checking.

### Adolescent involvement in this study

Two adolescent coresearchers, CW and EB, both undergraduate psychology students with previous experience contributing to health research projects, were engaged at multiple stages of this study. CW contributed to the development of topic guides for interviews using findings from previous evidence syntheses, providing input on the phrasing and sequence of questions. Both CW and EB helped recruit young people by reaching out to more than 200 organizations working on youth health and requesting them to share the study recruitment flyer with their youth advisory groups. They also collated the contact information of researchers listed as corresponding authors on relevant papers included in previous evidence syntheses and reached out to these authors to invite them to participate in the study. They were not involved in data collection due to the timeline of data collection clashing with their exam schedule. At the analysis stage, CW and EB both independently coded four interviews. They were engaged in the analysis process as a learning exercise for them and to share their unique insights in the interpretation of the results. They provided input on the themes by contributing to the development of codebook, and initial write‐up of themes and subthemes. Additionally, they cowrote the Introduction and Methods sections of this manuscript, as well as reviewed all sections of the manuscript, providing valuable input. In recognition of their crucial role throughout the project and in line with International Committee of Medical Journal Editors (ICMJE) guidelines, they are coauthors of the paper.

## RESULTS

**FIGURE 1:**
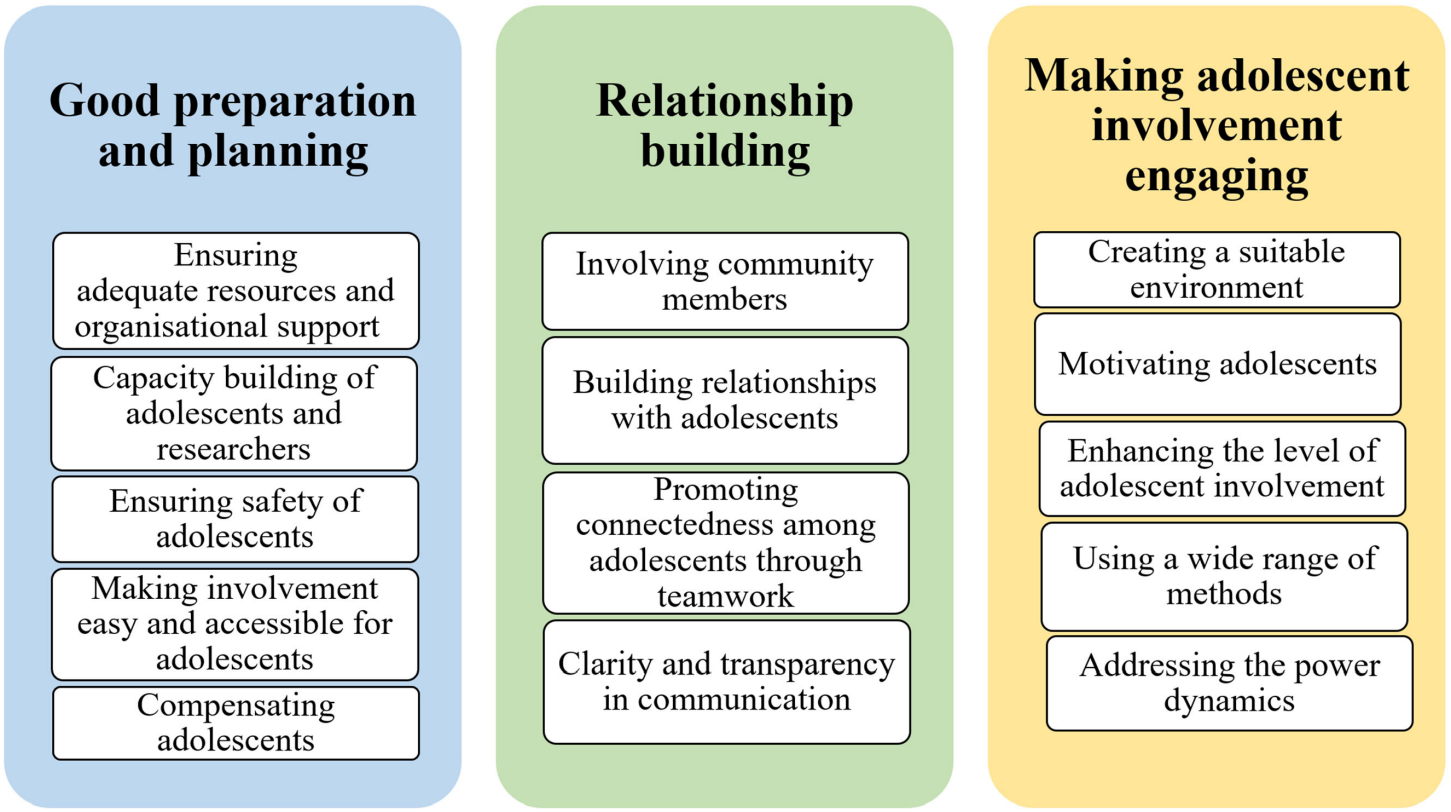
Themes identified from interviews with adolescents and researchers.

Although the interviews with adolescents and researchers were analyzed separately, we identified three themes in both sets of interviews, as discussed above. These three themes describe the strategies for addressing the barriers to meaningful adolescent involvement in health research (Figure 1).

### Good preparation and planning

This theme is centered around the importance of thorough planning and preparation to facilitate the meaningful involvement of adolescents in health research. It highlights the need for researchers to anticipate and address various aspects related to adolescent involvement in advance, to mitigate common barriers. These include ensuring the provision of adequate resources and organizational support, building the capacity of adolescents and researchers, making involvement easy and accessible for adolescents, compensating adolescents for their time, and planning to ensure their safety in the process.

Several measures need to be taken to ensure the provision of adequate resources and ***organizational support** for adolescent involvement*. Raising awareness plays a crucial role in gaining support, particularly in institutions unfamiliar with youth involvement. This includes educating ethics committees and senior staff: “*Quite a lot of background work needs to be done with those folks before starting*” (Isabella, researcher). Raising awareness can take various forms, such as workshops or seminars to “*chip away at people's mindsets and build the authenticity of youth engagement*” (Sunaina, researcher). To ensure organizational buy‐in, researchers also need to advocate for adolescent involvement by showcasing its impact, as stated by Sophie (researcher): “*You have to promote what's changing as a result of the input … … you've got to make it as if it's going to be virtually impossible to do it without [adolescent] involvement.*” Similarly, identifying key allies when involving adolescents in an unsupportive context or organization can also help: “*identifying one or two people within the decision making or managerial senior leadership, who are more likely to be swayed in your direction …. get them on board …. to get your foot in the door*” (Sunaina, researcher).

Researchers and adolescents further highlighted the need to ***plan for resources*** when budgeting. Researchers repeated that it was essential to consider adolescent involvement at the budgeting stage and add adequate amount of funds needed to involve adolescents: “*The biggest mistake is to underfund ‐ to ask for too little money ‐ because then you are quite likely to do something that is not very good if you haven't got the resources*” (Isabella, researcher). For adolescents, having adequate funding to provide them access to necessary equipment was also critical, “*You obviously cannot do research when you don't have maybe a laptop or a computer system to input your data and view the work progress.*” (Ekwoge, adolescent advisor).

When there is limited funding available, both researchers and adolescents highlighted the need to find alternatives, such as scaling down adolescent involvement or seeking input from existing advisory groups, “*It would be really useful to kind of have a standing group of young people as advisors … that you could go to initially, to prove your method or your proposal and that's centrally funded by your university*” (Siobhan, researcher). Adolescents suggested addressing limited resources by using alternatives to financial compensation to adolescents, such as “*If your project funds don't allow a stipend, then you [can] kind of invest in the person's capacity building, [such as] running courses…for free.*” (Aarti, adolescent consultant and coresearcher).

When planning for adolescent involvement, researchers should also establish realistic timelines. Adolescents also emphasized this point, saying that researchers “*should be able to set realistic time frames for [adolescents] to accomplish that project*” (Abena, adolescent consultant and collaborator). This may involve negotiating with funders, “*We need to say no to projects that are very short in timeline. I mean, if donors can't find anybody who's going to do it in a very short time*” (Reha, researcher).

Researchers need to ***build their capacity to engage adolescents and adolescents' capacity to contribute to the research process***. Researchers indicated that their own capacity building cannot be always gained from reading guidelines and resources and needs to be experiential: “*Researchers who don't have previous experience of adolescent involvement should be trained through learning by doing. Learning from guidance cannot replace what you learn by doing and how you build some of those skills in the field.*” (Charlotte, researcher). Additionally, some researchers highlighted the need for formal trainings of researchers in the form of research seminars, research modules, online courses, or informally by researchers sharing their learnings across disciplines. Adolescents suggested that researchers' training should focus on changing researchers' perceptions of young people's abilities; for example, Akua (adolescent consultant and coresearcher) said researchers, “*have to learn to understand that being young doesn't mean you don't have what it takes to do what the older people are doing.*” This also includes learning to set reasonable expectations from adolescents. Researchers also indicated this, saying: “*Researchers look down on the young people. We need to really learn to get away from this*” (Juma, researcher). Researchers recommended that their training should prioritize building skills in engaging adolescents, “*If you're involving young people, you need to develop some youth skills so that you are getting some in depth comments and not just sort of top‐of‐mind things that are not very deep and rich and helpful*” (Zoe, Researcher).

Reflecting on the capacity building of adolescents, adolescents emphasized that it is the responsibility of researchers to ensure adolescents are properly trained. Researchers indicated that capacity building of adolescents should focus on introducing them to research, while adolescents suggested that their training should be on different aspects such as research methods, public speaking, or building confidence. Both researchers and adolescents agreed that researchers should avoid making assumptions about adolescents' skill sets before training. For example, Daan (adolescent advisor) stated that researchers should, “*ask what kind of training youngsters would like or what do they need. … Why would you put your energy and time into trying to brainstorm solutions if you can just ask youngsters what they need?*” Similarly, Ochieng (researcher) said, “*I think there's that aspect of generally just assuming that these young people should know these things already.*”

Researchers also need to plan for ensuring the ***safety of adolescents during the involvement process***. While discussing the ethics of adolescent involvement, researchers proposed several measures to improve the current ethics review procedures for projects involving adolescents in research. This included “*training ethics committee members*” in order to “*avoid this protection versus participation approach to ethics procedures*” (Sophie, researcher). Discussions with ethics committees should also involve adolescents: “*I think it's important getting young people to speak up as well and get them at the table with those ethics committee members*” (Sophie, researcher). Some researchers indicated that there was no need for ethics approval for adolescent involvement, “*If it's public involvement, you don't need ethics committee approval*” (Emily, researcher) whereas others believed that this was essential.

Ensuring the safety of adolescents starts with protecting their rights, including their right to participate, right to privacy, right to be safe from harm, and their right to be respected. While discussing adolescents' right to participate, Emily (researcher) shared, “*It is important to involve vulnerable, marginalised young people ethically. But I think quite often some people can be more worried about protecting young people than enabling them to participate.*” Regardless of ethical clearance, adolescent involvement should be ethical in practice. At the organizational level, this can involve the development and implementation of policies and plans to protect adolescents, such as a youth protection policy, antiharassment training, and risk management and safeguarding plans for adolescents as reported by the participants. These can include quite broad policies like a “*child protection policy in the organisation*” (Andrei, researcher) or more specific protocols such as “*hav[ing] to be in a public place, you can't be traveling alone in a vehicle with a child*” Sunaina (researcher). Even aside from specific policies, researchers can provide support to adolescents and establish safe spaces for them. This was echoed by adolescents such as Emily, who said: “*make that safe space by asking youth if there is anything that we can do to make this space more comfortable and safer for you*.” Adolescents emphasized the importance of receiving adequate information about how their safety will be ensured. For example, Emily (adolescent consultant and advisor) stated “*if this study is anonymous, make sure that's clear beforehand. Because I know for me, sometimes I can be hesitant to share about topics because I just don't know what's happening with the research.*”

Another aspect of adolescent involvement that researchers should plan for is ***making research involvement easy and accessible for adolescents***. This includes ensuring that meeting venues are accessible and arranging transportation for adolescents to facilitate their participation. Regarding transportation, participants stressed the need to cover adolescent travel expenses or providing alternative options. This could include “*organis[ing] a bus for us so that it takes us from wherever we are coming from to the place*” (Abena, adolescent consultant and collaborator). To make their involvement easy for adolescents, researchers need to manage young people's competing demands and workload, as reported by both researchers and adolescents. Understanding adolescents' schedules is critical, as is flexibility should the adolescents become overwhelmed. Kadiatu (adolescent advisor) explained that researchers should, “*Check the kind of schedule that [adolescents] have. So, if they feel burdened, then substitute that particular young researcher with another researcher that has this free time to work within that space.*”

Researchers also need to simplify research involvement procedures for adolescents by involving adolescents in the process. Aarti (adolescent consultant and coresearcher) described her experience of this:

*One thing that really helped me was that my supervisor would ask me, ‘What are the kind of tasks that you're interested in doing? And what is your skill set?’ And based on that, she would design tasks for me that were suited to my interests and skill set.*

Researchers should also plan to ***compensate adolescents for their time and contributions***. This can include acknowledging young people, building connections for adolescents, supporting their roles as community leaders, providing further opportunities for participation in research projects, offering financial compensation, providing module credits, and building their capacity. Adolescents such as Kwama (consultant and advisor) indicated that they needed to actively ensure that they were acknowledged for their work: “*If I'm not getting credit for the work I'm doing, then I have to ask the researcher or whoever is in charge, ‘Why am I not getting the credits for my work done?*’” Both adolescents and researchers emphasized that compensation should be discussed in advance, with clarity about the specific form and level of acknowledgement. Ekwoge (adolescent advisor) stated the following:

*“Before you start the research work, it's also very important [for researchers] to explain to [adolescents] that, ‘Okay, in the end, I am going to acknowledge you or I'm going to pay you to do this work,’ so [adolescents] know what they are doing.”*

### Building relationships to engage adolescents

Our second theme is relationship building; researchers and adolescents both describe relationship building as a crucial part of addressing barriers to adolescent involvement in health research. This involves engaging community members, developing trust and respect‐based relationships with adolescents, promoting teamwork among adolescents, and being transparent in communication with all stakeholders to build strong relationships.

Building relationships with stakeholders through ***community involvement*** is essential for successful adolescent engagement. Researchers emphasized partnering with local organizations to facilitate adolescent involvement, indicating that researchers “*should work with an organisation because they have that relationship with youth, and then you're coming in through an already trusted relationship*” (Sophie, researcher). When working with community members or organizations, researchers indicated the need to “*be clear about the benefits to the organisation*” or community and “*what the greater impacts could be of being involved in that research*” (Olivia, researcher). Researchers and adolescents shared various strategies for engaging communities, such as holding town meetings and demonstrating a long‐term commitment to their involvement: “*Researchers have to be really dedicated to the methods and principles of community‐engaged research. For this, there needs to be a long‐term commitment to community involvement. Otherwise, it's a tokenistic thing to get funding*” (Emma, researcher).

Researchers and adolescents emphasized the importance of ***building relationships between adolescents and researchers*** and shared different strategies for this. Researchers suggested getting to know adolescents by spending time with them and using icebreakers. Adolescents similarly indicated the importance of these activities, with Charlotte saying: “*When [researchers] do icebreakers and play little activities with us, that really sets a friendly mood. It gets you familiar with the people you're working with, and makes the environment much easier to be able to talk in.*” Researchers and adolescents also shared that researchers need to value adolescents' input to build their trust in researchers. Anjali (researcher) explained, “*I would change something in front of them. If they said that this question can be asked in this manner, I would immediately type it up and change it to build trust.*” Adolescents further recommended that researchers accommodate adolescents' needs to build trust‐based relationships with them. For example, Emily (adolescent consultant and advisor) said, “*ask from the start about accommodations, it helps to kind of make sure that it is discussed beforehand if there is a youth engagement specialist or just having resources like mental health resources available to youth from the start*.”

Researchers and adolescents highlighted certain factors that can facilitate relationship building with adolescents. First, researchers and adolescents shared that connecting with adolescents through people and organizations with whom they already have a relationship is helpful: “*You need to work with people from that community. So that it's not like, who's this strange researcher approaching me? It's someone that they know that you're collaborating with*” (Zoe, researcher), which was echoed by adolescents. Second, allocating adequate time to build relationships with adolescents is critical. The third key component required for relationship building with adolescents is ensuring that the researchers have the needed capacity. Adolescents indicated that researchers should be friendly and passionate in order for adolescents to build trust‐based relationships with them. Ayan (peer educator and coresearcher) said, for example, “*If you show the people that you're dedicated, passionate, and energetic, the trust will just increase*.” Adolescents also suggested having a skilled mediator for relationship building, “*I think having a middle person there, like a youth engagement specialist is something that makes a big difference there just because there is someone who can kind of help to build that connection between researchers and youth*” (Emily, adolescent consultant and advisor).

Researchers and adolescents highlighted the key role of *teamwork among adolescents* in addressing barriers to adolescent involvement. Researchers can pair up adolescents to work together or divide them into groups based on their age range. Researchers and adolescents shared the practical value of pairing up adolescents to work together: “*The best way to talk to young people is to use a young person, because they understand each other, they understand the language, it's just easy*” (Thabo, adolescent peer educator). In team activities among adolescents, researchers recommended paying attention to the group dynamics: “*Think about gender and how adolescents feel about each other. If you see there are hierarchies and there's kind of horrible teenage stuff going on, then terminate it and split the group up a different way*” (Charlotte, researcher).

*Clarity and transparency in communication* with adolescents and other stakeholders are the building blocks of developing strong relationships with them. For example, when collaborating with an adolescent health organization to recruit adolescents, researchers should clearly define their target population and provide detailed criteria for the adolescents to ensure diversity. In terms of communication with adolescents, researchers and adolescents agreed that clarity is needed from the start about roles and required commitment, explanation of compensation for participation, and a clear description of how their feedback will be incorporated. Researchers and adolescents indicated that there should be ongoing communication with adolescents, and they should be kept updated about the research process. Daan (adolescent advisor) indicated that researchers should “*share with us what are you doing with our feedback and input and what the next steps are that you are taking*.” As part of this ongoing communication, researchers should seek adolescents' feedback on their involvement. Chukwu (researcher) suggested that this feedback could be sought in a few different ways: “*Give them an opportunity to write a blog about experiences, like, ‘How did it go? What worked and what didn't work?’ You can also try anonymous surveys as well.*” When researchers are giving adolescents feedback, it should be constructive in nature, and in line with their age, it should not be “*harsh*” and should instead “*highlight the strengths as well as improvement areas that would help them feel more encouraged*” (Aarti, adolescent consultant and coresearcher).

Researchers and adolescents suggested employing various methods to communicate with adolescents, such as engaging in dialogues and using different platforms to communicate. These could be formal such as debrief sessions or could be more informal conservations between the researchers and adolescents. Researchers proposed using various types of documentation to promote clarity in communication with adolescents, including contracts and agreed terms of reference. Adolescents conversely preferred informal methods of communication such as direct messages and WhatsApp groups. They suggested using a wide range of communication methods, indicating that researchers should “*consider what actually works best for youth. Sometimes texting is more accessible, whereas for other things, like maybe recurring meetings, email can help us stay on top of that with calendar invites*” (Emily, adolescent consultant and advisor).

### Making adolescent involvement engaging

To address barriers to adolescent involvement, in our third theme we find that it is essential to make the process comfortable and engaging. Researchers can accomplish this by creating a suitable environment, motivating adolescents, increasing their representation, using innovative and interesting methods, and addressing power imbalances.

Adolescents emphasized the importance of researchers making research involvement engaging by fostering a ***suitable environment for them***. Where an environment is “*friendly for young people*” that “*makes it easier for people to speak up*” (Nomvula, adolescent consultant and advisor). Flexibility is a crucial part of creating a conducive environment for adolescents, whose availability and level of involvement is unlikely to be static throughout a project. This flexibility should include allowing adolescents “*to leave the research anytime [they] want*” (Kofi, adolescent coresearcher) or “*giving them more time to come or allowing them to submit their projects from home*” (Fatima, adolescent collaborator). Because of their varied commitments and life changes, it is essential for researchers to accommodate them and support them: “*Ask us how we're feeling. Like, if we're feeling happy, uneasy, confident, unconfident, and then ask privately, what's wrong? Is there anything we can do to help?*” (Charlotte, adolescent consultant and collaborator). Researchers explained that they should create an atmosphere that enables adolescents to share their voices and opinions. This can be achieved by “*being an advocate for youth's voices*” and by being “*a good facilitator by listening to everyone. If someone is from some minority and has experiences different to the majority of the group, maybe they won't want to speak up. So be aware of that, and include them*” (Layla, researcher).

Researchers reported the need to ***motivate young people*** to make research engaging for them. They explained this can be achieved by understanding what matters to adolescents. For example, a researcher Olivia stated the following regarding motivating adolescents:

“*We offer vouchers and kind of recognition, and those are sort of standard things. But are they really enough? When you speak to youth about what matters to them, it's about what difference they're going to make, feeling part of something, feeling it's really helping them as a person, and I don't think we tap into those to motivate people.*”

Adolescents suggested framing research so that it addresses their priorities, including highlighting the benefits of involvement for adolescents. This can include, “*tell[ing] them the benefits of being involved, motivate[ing] them with that and they will be encouraged to be a part of it*” (Kadiatu, adolescent advisor).

Another way that researchers can make research involvement more engaging for adolescents is by ***enhancing the level of their involvement in the process***. Both researchers and adolescents suggested that this can be accomplished by providing more opportunities for adolescents to be meaningfully involved in the project. While not every adolescent wants to be involved in all components of a research project, many want to be actively engaged and heard throughout. It can be useful to allocate dedicated spaces for adolescents in projects. An essential part of enhancing the level of adolescent involvement includes actually incorporating their voices and suggestions, and “*actually act[ing] on what they tell us*” (Charlotte, researcher), then “*let[ting] them see it in [the] work*” (Akua, adolescent consultant and coresearcher). Where it is not possible to act on adolescents' inputs, researchers should “*tell them why we didn't act on it. So, they know that we do value what they're saying*” (Charlotte, researcher). While it is useful to involve the perspectives of multiple stakeholders, in these cases it is important to ensure that adolescent voices are given primacy:

*“We can have different perspectives represented in our research. They don't all have to agree on a nice solution and sort of a single answer. And if it isn't a single answer, if it's like young people say this and parents say that, then actually saying there are quite fragmented views”* (Zoe, researcher)

To make involvement engaging, researchers and adolescents suggested using a wide range of flexible ***methods*** with adolescents. This ensures that “*there are different ways for adolescents to contribute*” (Lily, researcher) as there is “*no one right method*” (Abiy, researcher). Researchers advocated for the use of “*innovative*” methods, such as “*focus groups, interviews, arts and crafts, drawing exercises, role plays, or drawing chits and doing acting*” (Aditi, researcher). Adolescents also recommended methods that make research “*more engaging and interactive*” (Abena, adolescent consultant and collaborator), such as incorporating “*writing, graphics*” (Nomvula, adolescent consultant and advisor) or “*practical pieces*” (Emily, adolescent consultant and advisor). Researchers discussed the importance of being flexible in the selection of methods: “*Try to be flexible, try to give choice, not to stick to your agenda. If a method is clearly not working, go with the flow.*” (Isabella, researcher). Being flexible in the choice of methods also involves seeking young people's input in the selection of methods, “*Researchers should just let them choose the methods. And this is a scary thing for many researchers, I think, because again, going back to the ethics, we're told that we have to have prescriptive methods*” (Sara, researcher).

*Addressing the power dynamics* between researchers and adolescents is crucial for making the involvement process engaging for adolescents. To address these imbalances, firstly, researchers should be mindful of existing power dynamics and of their own attitudes and biases that contribute to power dynamics. Amelia (researcher) shared her experience of identifying these factors:

*“I think they struggled to be completely honest with me because of the power dynamic. I think they want to seem like they're doing a good job, so maybe they're worried … I would think they weren't doing it well.”*

Addressing these imbalances then entails respecting and valuing adolescents and making the value of genuine collaboration known to the adolescents. Anjali (researcher) said that she does this by continually “*driving home the point that, ‘I'm here to learn from you, because you have the experience, I want to hear about it from you*’.” By involving adolescents “*in as many of the decisions as possible*” (Amelia, researcher) and “*every part of the research project*” (Saoirse, adolescent coresearcher), the power imbalance can be reduced. Additionally, adolescents recommended equal treatment of researchers and adolescents.

*“I should feel that whatever I say won't be looked down upon because everyone is equal. So, I feel like power dynamics will reduce because … it's a room of people coming to work, trying to find solutions, to address the problem.”* (Nomvula, adolescent consultant and advisor)

Researchers also suggested addressing these imbalances by changing their working style to be less formal and engaging in exercises which show that “*everyone is different but important*” (Aditi, researcher). Adolescents indicated that researchers should meet them where they are at, highlight that lived experience is valuable, and show appreciation for the things that adolescents do.

## DISCUSSION

This study highlights which best practices of adolescent involvement can be used to address the commonly experienced barriers to adolescent involvement in health research from the perspectives of both adolescents and researchers across a wide range of contexts. We identified three overarching strategies necessary for addressing these barriers: adequate preparation and planning for adolescent involvement, building relationships, and making research involvement engaging for adolescents. Careful planning and preparation involve ensuring adequate resources for adolescent involvement, gaining organizational support, and making involvement accessible, safe, and fairly compensated. Both researchers and adolescents also benefit from hands‐on learning and a shift in how adolescents' capabilities are viewed. Building trust and relationships with adolescents and community are equally important, which can be achieved by partnering with local organizations, taking time to engage adolescents, and keeping communication open and transparent. Lastly, to keep adolescents engaged, it is crucial to create a flexible, supportive environment, use creative and interactive methods, and address power imbalances by treating adolescents as equals and involving them in decision‐making.

The broader themes for these strategies have previously been highlighted in the literature from researchers' perspectives. For example, a review by Larkins and colleagues emphasized the need to adequately plan and prepare for adolescent involvement by understanding the context, building connections, and planning for resources (Larkins et al., 2021). Similarly, previous work has suggested that successful involvement depends on building relationships with adolescents during the involvement process (Brady et al., 2018) and involving community members (Liebenberg et al., 2017). Furthermore, the importance of making involvement engaging for adolescents has also been suggested in some studies (Larkins et al., 2021). Our study builds on this previous research by highlighting how different strategies within these broader principles are key to addressing most of the commonly experienced barriers to adolescent involvement in health research. By including insights from both researchers and adolescents across diverse contexts and settings, we provide a more comprehensive understanding of how to effectively address the barriers to adolescent involvement in health research. This dual‐perspective approach ensures that the strategies we highlight are practical, inclusive, and adaptable, and directly informed by the experiences and views of adolescents themselves. For example, one of the most commonly reported barriers in the literature is limited resources (Warraitch, Lee, Bruce, et al., 2024). While previous studies have emphasized the importance of planning for adequate resources (Wilson et al., 2020), our study provides insights into what is required for the successful planning of adequate resources and suggests alternatives for involving adolescents effectively in low‐resource settings. Similarly, while the need for planning to ensure the safety of adolescents is frequently highlighted in the literature (Larkins et al., 2021), there are limited recommendations on improving the ethics review procedures and balancing the aspects of protection versus participation in safeguarding adolescents. Some studies have previously reported their challenges with ethics committees and have shared their recommendations, such as seeking exemptions for the anonymity of youth involved as research partners to enable them to claim ownership of their work (Yanar et al., 2016). Additionally, in one study, the authors submitted a separate application for involving youth as coinvestigators to conceptualize their involvement as equal partners and divided the application into smaller chunks to facilitate the ethical review process (Abraczinskas et al., 2022). Our study explores the mitigation of similar challenges faced by researchers in different settings and adds their perspectives.

Furthermore, most of the previous research highlighting these principles has been from the perspective of researchers and other professionals (Das et al., 2020; Faithfull et al., 2019; Hawke et al., 2020; Oruche et al., 2014; Radez et al., 2021; Spielvogle, 2011; Wadman et al., 2019). This prioritization of adult viewpoints on addressing the challenges to adolescent involvement is linked to “adultism,” which is the belief that adults are superior to young people and, by default young people are inferior (Shier, 2012). Participants in our study identified adultism as a barrier to adolescent involvement in their projects, describing it as negative attitudes and doubts about adolescents' competence. A recent study also found that adults are less likely to endorse youth's right to participate as compared to other rights in the UN Convention on Rights of the Child (Bourke et al., 2020). Youth Participatory Action Research (YPAR) has been suggested as an effective approach to counteract adultism by positioning youth as experts in their own lives (Kennedy, 2019). However, adolescents in our study indicated that adultism cannot be simply addressed by involving youth as experts in their lives but requires further strategies to equip them with the resources and support needed to tackle the adult‐centered notions. This includes building adolescents' capacity to prepare them for their roles to address adultism, which is in line with other studies highlighting the need for sharing “knowledge and skills with youth” to address researchers' perception of youth not being competent to contribute to research (Corney et al., 2022). Moreover, Conner et al. (2016) warned that YPAR can perpetuate adultism if it is tokenistic or used to lend credibility without genuinely integrating youth input (Gaventa & Cornwall, 2015). To address this, changes are needed at the funder, organizational, community, and researcher levels. Some of these changes needed to address adultism include changing research norms, engaging in reflexivity, and using a youth‐centered design (Bettencourt, 2020).

This study further contributes to the literature by incorporating the unique insights of adolescents on the most effective ways to address the barriers to adolescent involvement. There was a significant overlap in the broader themes of the strategies proposed by adolescents and researchers. This is noteworthy, considering the varying nature of the barriers experienced by each group. However, within the overarching categories of proposed strategies, researchers often approached adolescent involvement from a professional standpoint, mostly focusing on systemic issues and proposing tangible actions for researchers to tackle the barriers at a higher level. In contrast, adolescents' suggestions were often more rooted in their lived experiences and usually stemmed from the personal challenges they face when engaging in research activities, resulting in more individualistic rather than system‐level suggestions. The convergence of similar strategies from both professional and lived experience perspectives strengthens the validity and significance of these strategies in engaging adolescents in health research.

Many of the proposed strategies were recommended to address several types of barriers. For example, ensuring adolescents' anonymity and confidentiality was recommended to address several related issues: It reduces their hesitation to participate by providing a sense of safety, protects their well‐being by giving them control over their personal data, and meets the ethical standards set by institutional review boards. By implementing such strategies, researchers can simultaneously overcome various challenges, such as youth's reluctance to participate, safety concerns, and ethical considerations.

Similarly, strategies for building relationships with adolescents and community members were suggested for addressing multiple barriers. These included structural barriers at the organizational level (e.g., by identifying allies within organization and partnering with community members to take gatekeepers on board), issues of limited resources (e.g., hiring someone dedicated to youth engagement in the organization when researchers have limited time for youth engagement), barriers related to researchers' competence and skills to engage adolescents (e.g., by partnering with adolescents' trusted advisors to seek guidance on adolescent involvement), challenges in retaining adolescents in research (e.g., by building rapport and trust with youth), and addressing power dynamics (e.g., by making them feel more involved and valued). This is in line with other studies suggesting that individual strategies can address multiple barriers simultaneously as the various challenges to adolescent involvement are also interconnected (Warraitch, Lee, Bruce, et al., 2024a).

The proposed strategies for adequate preparation and planning, building relationships, and making research involvement engaging for adolescents are interdependent and mutually reinforcing in promoting meaningful adolescent involvement. Adequate preparation and planning lay the groundwork for fostering of strong relationships and an engaging research involvement process (Costello & Dorris, 2020; Das et al., 2020; Larkins et al., 2021). Strong relationships, by allowing for the incorporation of the perspectives and needs of adolescents and community stakeholders, can enhance the preparation and planning process (Rouncefield‐Swales et al., 2021). As highlighted by the participants in our study, a more engaging research process strengthens adolescent–researcher relationships and fosters adolescents' sense of ownership and commitment to the research process. In turn, strong relationships support sustained engagement in the research process (Brown et al., 2020; Hackett, 2019; Langdon et al., 2016; Roxas et al., 2017). These strategies can be applied to address the most commonly experienced barriers to adolescent involvement across various youth involvement methods, including youth participatory action research, community‐based participatory research, and involving youth as consultants, collaborators, or coresearchers. The recommended strategies should be implemented at all stages of the research process. For example, good preparation and planning are not limited to the preengagement stage. Researchers must continuously reflect on the involvement processes and adapt the procedures, necessitating ongoing planning (Larkins et al., 2021; Pavarini et al., 2019). Similarly, relationship building should commence from the initial phase of adolescent involvement, through community involvement and partnering with local gatekeepers, and needs to be sustained long term (Das et al., 2020). Ensuring engaging research involvement requires planning from the project conceptualization stage through to the completion of the research project.

Collaborative action is required from stakeholders at different levels to translate these strategies into practice and thus to address the barriers to adolescent involvement (Warraitch, Lee, Bruce, et al., 2024). For example, organizational leaders should allocate resources and foster a culture that values youth engagement (Faithfull et al., 2019) in order to facilitate researchers in implementing these strategies. Ethics committees should work with researchers to ensure the involvement procedures incorporate these recommendations. Researchers must actively involve and support adolescents in research processes by implementing these actions. Community partners should provide access to diverse youth populations and collaborate with researchers in relationship building (Das et al., 2020; Wilson et al., 2020). By working together, these actors can overcome the barriers to adolescent involvement and create meaningful opportunities for adolescent participation and empowerment. These strategies can also be applied to address the barriers to adolescent involvement in other areas of research, in addition to health research. For example, they are also relevant for enhancing meaningful adolescent involvement in educational research (Bourke et al., 2022; Mallon et al., 2023; Martinez‐Vargas et al., 2024).

The findings from this study should be interpreted in light of a few limitations. First, we only interviewed adolescents and researchers who were fluent in English. Second, most of the adolescents involved were 18 or older. Third, due to an extensive list of challenges to adolescent involvement identified from the literature, we asked different questions to various participants to explore mitigation strategies for all identified barriers. This may have prevented us from achieving data saturation within 50 participants. Fourth, we were unable to ensure a balance in the number of participants recruited from low‐, middle‐, and high‐income countries, potentially resulting in greater representation of views from certain contexts. For example, we had a greater representation of adolescents from low‐ and middle‐income countries and a greater representation of researchers from high‐income countries. Fifth, since most of the youth were involved in multiple capacities and roles in different projects, it was not possible to segregate the analysis or recommendations based on the different roles they had in different studies.

## CONCLUSION

This study identified key mitigation strategies for addressing commonly experienced barriers to adolescent involvement in different areas of health research such as physical and mental well‐being and ill‐health, sexual and reproductive health, bullying and violence prevention, and more. These interdependent and mutually reinforcing strategies form a comprehensive approach to maximize the effectiveness of initiatives aimed at fostering adolescent involvement in health research. This study establishes these strategies and their encompassing actions as best practices of adolescent involvement from the perspective of both adolescents and researchers and underscores the importance of incorporating these strategies at all stages of the research process to overcome the challenges to adolescent involvement in health research.

## FUNDING INFORMATION

AW received the Ussher Fellowship from Trinity College Dublin to support her PhD research, including this study. AB received a PPI Research grant to involve youth in this study from Dublin City University. The funders did not play any role in the design of this study.

## CONFLICT OF INTEREST STATEMENT

The authors declare that they have no competing interests.

## PATIENT CONSENT STATEMENT

Written informed consent was obtained from all participants aged 18 and above, from the parents of adolescents under the age of 18 years, and written assent was sought from adolescents under the age of 18 years.

## Data Availability

The data that support the findings of this study are available on request from the corresponding author. The data are not publicly available due to privacy or ethical restrictions.
